# Mesenchymal stem cells enhance selective ER-phagy to promote α-synuclein clearance in Parkinson’s disease

**DOI:** 10.1093/stcltm/szaf019

**Published:** 2025-06-10

**Authors:** Ji Eun Lee, Kyu Won Oh, Jin Young Shin, Yeon Ju Kim, Seung-Jae Lee, Phil Hyu Lee

**Affiliations:** Department of Neurology, Yonsei University College of Medicine, Seoul 03722, South Korea; Department of Neurology, Yonsei University College of Medicine, Seoul 03722, South Korea; Department of Biomedical Sciences, Neuroscience Research Institute, Seoul National University College of Medicine, Seoul 03080, South Korea; Department of Neurology, Yonsei University College of Medicine, Seoul 03722, South Korea; Department of Neurology, Yonsei University College of Medicine, Seoul 03722, South Korea; Department of Biomedical Sciences, Neuroscience Research Institute, Seoul National University College of Medicine, Seoul 03080, South Korea; Convergence Research Center for Dementia, Seoul National University College of Medicine, Seoul 03081, Korea; Neuramedy Co. Ltd., Seoul 04796, South Korea; Department of Neurology, Yonsei University College of Medicine, Seoul 03722, South Korea; Department of Biomedical Science, Yonsei University College of Medicine, Seoul 03722, South Korea

**Keywords:** α-synuclein, ER-phagy, FAM134B, mesenchymal stem cells, NR4A1, Parkinson’s disease

## Abstract

Ample evidence suggests that α-synuclein (αSyn) accumulation in the endoplasmic reticulum (ER) leads to ER stress, resulting in neurodegeneration in Parkinson’s disease (PD). Selective degradation of accumulated αSyn through ER-phagy can alleviate ER stress and rescue neurodegeneration. In the present study, we investigated whether mesenchymal stem cells (MSCs) exert neuroprotective effects against PD by modulating ER-phagy. In a cellular model overexpressing αSyn specifically in the ER (ER-αSyn), co-culture with MSCs promoted ER-αSyn clearance through selective ER-phagy and also recovered cell viability. Injection of MSCs to an animal model using adeno-associated virus vectors to overexpress αSyn in the ER (AAV-ER- αSyn), also decreased the expression of aSyn in the ER and attenuated the dopaminergic neuronal loss in substantia nigra (SN) and denervation in striatum (ST), followed by functional improvement of motor deficits. In vitro screening identified that MSCs promoted family with sequence similarity 134 member B (FAM134B)-mediated ER-phagy via regulating transcription factor of nuclear subfamily 4 group A member 1 (NR4A1), and it underwent in vivo validation. This study suggests that MSCs modulate FAM134B-mediated ER-phagy under the regulation of NR4A1, promoting the clearance of ER-accumulated αSyn in PD cellular and murine models.

## Graphical Abstract

**Figure:**
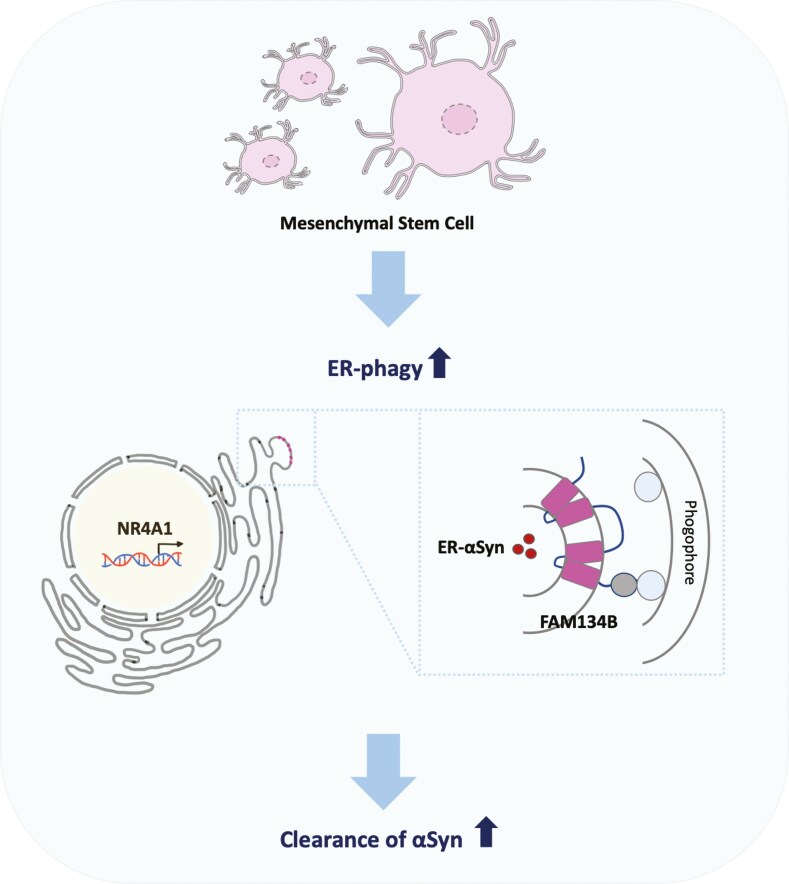
MSCs modulate FAM134B-mediated ER-phagy under the regulation of NR4A1, promoting the clearance of ER-accumulated αSyn in PD cellular and murine models.

**Significance statement**

This is the first study to investigate the involvement of nuclear subfamily 4 group A member 1 (NR4A1) in selective endoplasmic reticulum (ER)-phagy. Through the regulation of NR4A1, a family with sequence similarity 134 member B (FAM134B)-mediated ER-phagy enhancement occurs, subsequently promoting the clearance of α-synuclein (αSyn). The present study offers a promising therapeutic strategy for the clearance of ER-accumulating αSyn.

## Introduction

Parkinson’s disease (PD) is the second most common neurodegenerative disorder, characterized by motor symptoms such as rigidity, resting tremor, bradykinesia, and postural instability.^1^ Pathologically, PD involves the progressive loss of dopaminergic neurons in the substantia nigra (SN) and the presence of Lewy bodies, which are cytoplasmic inclusions primarily composed of aggregated alpha-synuclein (αSyn).^2,3^ Typically, αSyn exists in a monomeric form^4^; however, when it aggregates, it disrupts cellular homeostasis, including impairing lysosomal clearance systems.^5,6^

Among cellular organelles, the endoplasmic reticulum (ER) plays a crucial role in protein homeostasis. Accumulation of misfolded proteins within the ER triggers ER stress. Numerous studies have demonstrated the involvement of ER stress in PD. For instance, in a 6-hydroxydopamine (6-OHDA)-induced cellular Parkinson’s model, the ER stress marker C/EBP homologous protein (CHOP) was upregulated, accompanied by neurite degeneration and somal shrinkage.^7^ Conversely, CHOP knockout protected dopaminergic neurons in the SN in a 6-OHDA murine PD model.^8^ The dopaminergic system’s specific sensitivity to ER stress is evident, as deficiency in XBP1, a major transcription factor of the unfolded protein response, triggers the upregulation of ER stress response proteins in SN dopaminergic neurons but not in other brain regions.^9^ Recent studies also link ER stress to αSyn accumulation in the ER, with observations of αSyn oligomers and aggregates in murine models of synucleinopathy^10^ and human patients with PD.^11^ These accumulations manifest as chronic ER stress followed by neurodegeneration; however, treatment with the anti-ER stress agent Salubrinal attenuates these pathological signs and reduces αSyn accumulations in the ER.^10,11^ Therefore, modulating ER-accumulated αSyn and subsequent ER stress is a promising strategy for PD treatment.

Ample evidence suggests that deficient organelles and misfolded proteins are selectively degraded through autophagy.^12,13^ Selective autophagy is a cellular quality control network that involves several pathways classified according to the targets: mitophagy (mitochondria), lysophagy (lysosomes), aggrephagy (protein and RNA aggregates), xenophagy (intracellular pathogens), ER-phagy, pexophagy (peroxisomes), or ribophagy (ribosomes).^12,14,15^ This process requires specific receptors that bind to cargoes and LC3 on autophagosomal membranes. In the case of ER-phagy involving selective recognition of the ER, known receptors include FAM134B, RTNL3, ATL3, SEC62, CCPG1, and TEX264.^16-22^ Our previous study demonstrated that FAM134B is a significant contributor to αSyn clearance in cellular and animal models overexpressing αSyn in the ER (ER-αSyn). Conversely, knockdown of FAM134B resulted in significant loss of dopaminergic neurons in the SN and striatal postsynaptic neurons in a Parkinsonian mouse model.^23^

Mesenchymal stem cells (MSCs) have shown neuroprotective effects in various neurological disorders, mediated by cytotropic factors such as neurotrophic growth factors, chemokines, cytokines, and extracellular matrix proteins.^24-27^ Previous studies have demonstrated that MSCs contribute to the degradation of misfolded proteins by modulating autophagy.^28,29^ However, most studies on autophagy modulation have focused on macroautophagy, with a lack of research on the role of selective autophagy in regulating misfolded proteins. In the present study, we hypothesized that MSCs would promote αSyn clearance by modulating selective ER-phagy in parkinsonian models. Therefore, we evaluated whether MSCs exert neuroprotective effects through the modulation of ER-phagy in cellular and murine models of PD with αSyn accumulation in the ER.

## Materials and methods

### MSCs and PC12 culture

In this study, frozen vials of human bone marrow-derived mesenchymal stem cells (MSCs) were obtained from the Korean Cell Line Bank (South Korea). MSCs were maintained in low glucose Dulbecco’s Modified Eagle Medium (DMEM; HyClone) supplemented with 10% fetal bovine serum (FBS; HyClone) and an antibiotic mixture of 1% penicillin and streptomycin (P/S; Corning). The rat pheochromocytoma cell line, PC12, was maintained in high glucose DMEM (HyClone) supplemented with 10% FBS (GeneDEPOT) and 1% P/S. When cells reached over 80% confluency, they were trypsinized and subcultured. MSCs were kept under passage 10 to preserve their capacity and stemness,^30^ as a prolonged subculture and later passage reduce these qualities. Cells were cultivated in a humidified incubator at 37 °C and 5% CO_2_. To evaluate the effects of MSCs, they were cultured on the permeable membrane of a transwell insert, while PC12 cells were cultured at the bottom of a 6-well plate.

### Plasmid transfection

For transfection, we cloned αSyn into a vector containing the ER retention signal sequence (ER-αSyn) with Keima protein (ER-Keima-αSyn),^23^ and NR4A1 and empty plasmids were generously provided by Prof. Yong Jun Choi (Ajou University, South Korea).^31^ PC12 cells were seeded 24 hours before transfection and grown to 70%-80% confluence. Cells were transfected using jetPrime transfection reagent (Polyplus) according to the manufacturer’s instructions. To knock down NR4A1 in PC12 cells, small interfering RNA (siRNA) constructs (Bioneer) were purchased and tested for knockdown efficiency. siRNA transfection into PC12 cells was also performed using jetPrime transfection reagent according to the manufacturer’s protocol.

### Cell viability analysis

Cell viability was measured using the MTS cell proliferation assay (Promega). Right before the measurement, MTS and PMS were mixed in a 1 mL:50 µL ratio and incubated for 10 min. Then, 20 µL of the mix was added to each well. Plates were incubated at 37°C for 1 hour, and absorbance was measured at 490 nm using a spectrophotometer. All experiments were repeated at least 3 times.

### Western blotting

Equal amounts of total protein were separated by sodium dodecyl sulfate polyacrylamide gel electrophoresis (SDS-PAGE) and transferred to hydrophobic polyvinylidene difluoride (PVDF) membranes (GE Healthcare). Membranes were blocked in 5% skim milk in PBST and probed with the following primary antibodies: mouse anti-αSyn (Santa Cruz, sc-12767), mouse anti-actin (Santa Cruz, sc-47778), mouse anti-Keima (MBL, M182-3M), mouse anti-CHOP (Santa Cruz, sc-7351), rabbit anti-FAM134B (Proteintech, 21537-1-AP), rabbit anti-CCPG-1 (Proteintech, 13861-1-AP), rabbit anti-LC3B (MilliporeSigma, L7543), mouse anti-rab7 (Abcam, ab50533), rabbit anti-LAMP1 (Abcam, ab24170), and mouse anti-NR4A1 (Santa Cruz, sc-365113). As secondary antibodies, 1:5000 dilutions of horseradish peroxidase-conjugated antibody (GenDEPOT) were used. Antigen-antibody complexes were visualized with ECL solution (GenDEPOT). For quantitative analysis, immunoblotting band densities were measured using ImageJ software.

### Immunocytochemistry and Immunohistochemistry

PC12 cells were fixed and permeabilized using a 3:1 methanol and acetone mixture. The fixed cells were then rinsed three times with 0.1 M glycine in PBS. Brain tissues were fixed with 4% paraformaldehyde. Both the cells and brain sections were blocked with 0.5% bovine serum albumin in PBST. After blocking, the samples were incubated with the following primary antibodies: mouse anti-PDI (Invitrogen, MA3-019), rabbit anti-αSyn (Abcam, ab138501), mouse anti-LC3B (MBL, M152-3), rabbit anti-FAM134B (Proteintech, 21537-1-AP), mouse anti-TH (Sigma, T2928), and rabbit anti-dopamine transporter (DAT) (Sigma, AB1591P). Immunofluorescence labeling was performed using mouse anti-IgG Alexa Fluor-488 and rabbit anti-IgG Alexa Fluor-647 (Invitrogen). Cell nuclei were counterstained with 4′, 6-diamidino-2-phenylindole (DAPI, Invitrogen). The anti-TH and anti-DAT antibodies were detected with 0.05% DAB staining (Vector Laboratories). Immunofluorescence-stained samples were analyzed using confocal microscopy on a Zeiss LSM 700 confocal imaging system, while DAB-stained samples were analyzed using bright-field microscopy.

### Adeno-associated viral vector preparation

The plasmids for recombinant AAV (rAAV) vector production included the construct for the AAV8 serotype, the AAV transfer plasmid, and the pAdvDeltaF6 adenoviral helper plasmid. The rAAV serotype 8 expressing human WT-αSyn was driven by a mouse synapsin-1 promoter and enhanced by a woodchuck hepatitis virus posttranscriptional regulatory element. The virus was produced by the Korea Institute of Science and Technology (Seoul, South Korea). Animals were injected with 2 µL of AAV8 ER-αSyn (approximately 2.5 × 10^13^ genome copies per milliliter) into the right and left SN at a flow rate of 0.5 µL/min.

### Animal study

All animal experimental procedures were approved by the Institutional Animal Care and Use Committee of the Yonsei University Health System (no. YUHS-IACUC-2022-0031). Male C57BL/6J mice (5 weeks old) were acclimated in a climate-controlled room with a 12-hour light/dark cycle for one week prior to the experiment. At 6 weeks of age, the mice were randomly divided into 3 groups: Control group, AAV-ER-αSyn group, and AAV-ER-αSyn + MSCs treatment group. Briefly, the mice were anesthetized with isoflurane (Baxter), and the viruses were slowly injected into the SN (–3.1 mm posterior to bregma, ± 1.2 mm lateral to midline, and –4.3 mm ventral to the brain surface) using a stainless steel 26-gauge injection needle connected to a 1 mL microsyringe (Hamilton). The needle was left in place for 10 minutes before being slowly withdrawn. Three days after virus inoculation (postoperative day 3), mice in the MSCs treatment group received a tail vein injection of MSCs (1 × 10^6^ cells per 100 µL). Animals were sacrificed 4 d, 1 week, and 4 weeks after MSCs injection, respectively.

### Preparation of brain tissue

Upon completion of the animal experiment, mice were sacrificed, and their brains were collected. For immunohistochemistry, the mice were perfused with 4% paraformaldehyde. The brains were then harvested, post-fixed for 72 hours in 4% paraformaldehyde, and stored in a 30% sucrose solution at 4 °C for 1-2 d until they sank. Subsequently, 25-μm coronal sections were obtained using a cryostat. These sections were stored in a tissue storage solution (30% glycerol, 30% ethylene glycol, 30% distilled water, 10% 0.2 M PB) at 4 °C until needed. For western blot analysis, the striatum (ST) and midbrain were dissected, and total protein was extracted using RIPA buffer (50 mM Tris-HCl, pH 7.5, with 150 mM sodium chloride, 1% Triton X-100, 1% sodium deoxycholate, 0.1% SDS, 2 mM EDTA; Lugen Sci, Korea) with a protease inhibitor cocktail (Sigma).

### Behavioral tests

To assess motor function, coordination, and balance, mice were tested using the Rotarod apparatus (MEDAssociates). Prior to testing, the mice were trained to run on the rotarod at 20 rpm for 10 minutes daily for 5 consecutive days before virus injection. During the test sessions, the mice ran on the rotarod with increasing speeds from 5 to 50 rpm and at a constant speed of 30 rpm (with a cutoff time of 600 seconds). The latency time until the mice fell off the rotarod was recorded. For the pole test, we followed the protocol described in a previous study.^32^ Each mouse was placed on top of a vertical plastic pole (30 cm in height). On the day before testing, mice were allowed to descend from the top of the pole three times to become habituated to the apparatus. The total time it took each mouse to reach the base of the pole and place its front paws on the floor was recorded. Each mouse repeated the test 3 times. All mouse groups were randomized and performed blind to the experimenter.

### Quantitative real-time PCR

Total RNA was isolated from PC12 cells using TRIzol reagent (Lugen Sci) according to the manufacturer’s instructions. Equal amounts of RNA (approximately 1 μg) were reverse transcribed using a cDNA synthesis premix (Applied Biosystems). A master mix of the following reaction components was prepared to the indicated end concentration: 12.5 μL of 2X SYBR Green buffer, 2 μL of forward and reverse primers (10 pmol each), and 2 μL of DNA template (100 ng). Amplification conditions were as follows: initial denaturation at 95 °C for 2 minutes, followed by 40 amplification cycles of 95 °C for 15 s and 60 °C for 1 minutes for annealing and extension, respectively. Quantitative PCR experiments were performed using an Applied Biosystems (Thermofisher Scientific) machine. The quantitative real-time PCR reaction utilized 10 pmol each of the primers for rat NR4A1, FAM134B, and GAPDH (Table 1).

**Table 1.** PCR primers.

Primer name	Sequence
NR4A1-F	5′-GAA AGT TGG GGT AGT GTG CGA-3′
NR4A1-R	5′-GCT GGT TGC TGG TGT TCC ATA -3′
FAM134B-F	5′-TGG AGG AGC CTC AGT GAA AG-3′
FAM134B-R	5′-GTA GCT GAG AAT GAC CCC AGG-3′
GAPDH-F	5′-AGT GCC AGC CTC GTC TCA TA-3′
GAPDH-R	5′-GGG TTT CCC GTT GAT GAC CA-3′

### Statistical analysis

To evaluate differences among the experimental groups, Kruskal-Wallis nonparametric analysis followed by Dunn’s multiple comparison tests were used. Mann-Whitney U test was used to compare the 2 independent groups. Differences were considered statistically significant at *P* < .05. Statistical analysis was performed using SPSS v. 25 (Yonsei University).

## Results

### MSCs promote clearance of αSyn accumulated in the ER and exert neuroprotective effects in PC12 cells

**Figure 1.**
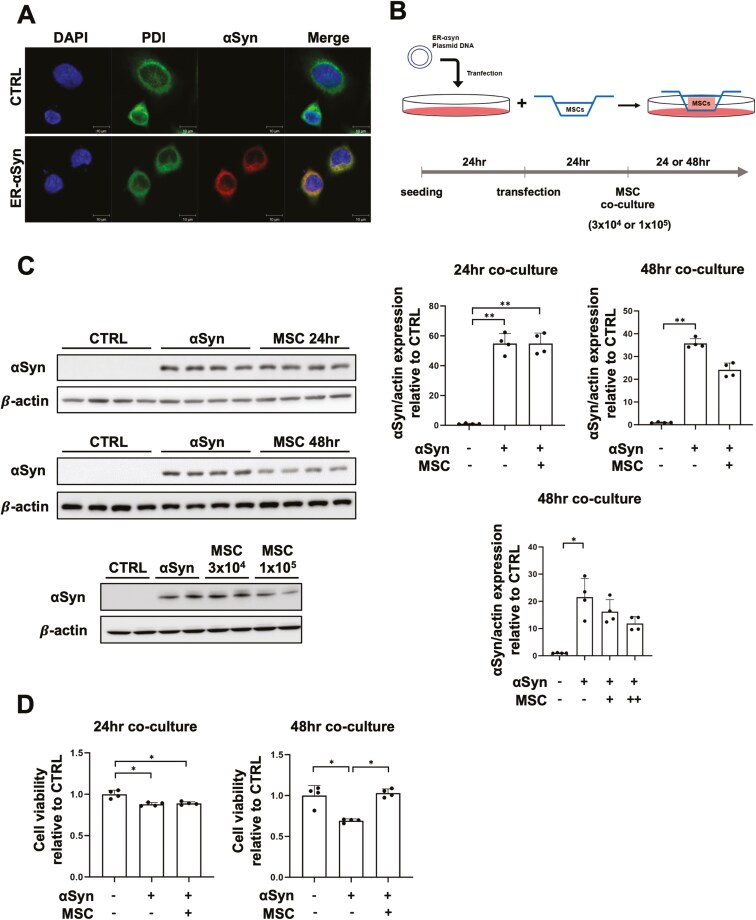
MSCs promote the clearance of αSyn accumulated in the ER and exert a neuroprotective effect in PC12 cells. (A) Immunostaining for αSyn overexpressed in the ER in PC12 cells. Scale bar, 10 μm. (B) Schematic illustrations and experimental design schedule. (C) Western blot analysis of αSyn in control, ER-αSyn, and co-culture with MSCs groups (n = 4 per group). Quantification of αSyn western blot analysis. 3 × 10^4^ MSCs= +, 1 × 10^5^ MSCs= ++. (D) Cell viability analysis in control, ER-αSyn, and co-culture with MSCs groups at 24- and 48-hour co-culture (*n* = 4 per group). Differences among conditions were evaluated by Kruskal-Wallis with Dunn’s test. Data are presented as mean ± SE. *P < .05, **P < .01, ***P < .001.

To investigate selective ER-phagy in an αSyn-overexpressing Parkinsonian model, we created a construct cloning αSyn into a vector containing the ER retention signal (ER-αSyn). Using an ER marker, we confirmed that αSyn exclusively accumulates in the ER (Figure 1A). We then cocultured MSCs with ER-αSyn overexpressing PC12 cells to determine if MSCs modulate αSyn accumulated in the ER (Figure 1B). To mimic co-culture environment, transwell system was applied to separate MSC and ER-αSyn overexpressing PC12 populations but to enable paracrine signaling. However, control or ER-αSyn overexpressing PC12 (used the term “αSyn” in figures) group was cultured alone. In our previous studies, the controls used in cellular experiments were cultured alone with no using co-culture system.^23,27,29,33-36^ Western blot analysis revealed that co-culture with MSCs decreased the expression of αSyn in the ER compared to the ER-αSyn group, with this effect being dose- and time-dependent (Figure 1C). The MTS assay demonstrated that cell viability was significantly reduced in the ER-αSyn group compared to controls, whereas co-culture with MSCs for 48 hour mitigated ER-αSyn-induced cell death (Figure 1D). Based on these results, a 48-hour co-culture period with 1 × 10^5^ MSCs was established for this study.

### MSCs promote clearance of αSyn in the ER and attenuate ER stress through modulating selective ER-phagy

**Figure 2.**
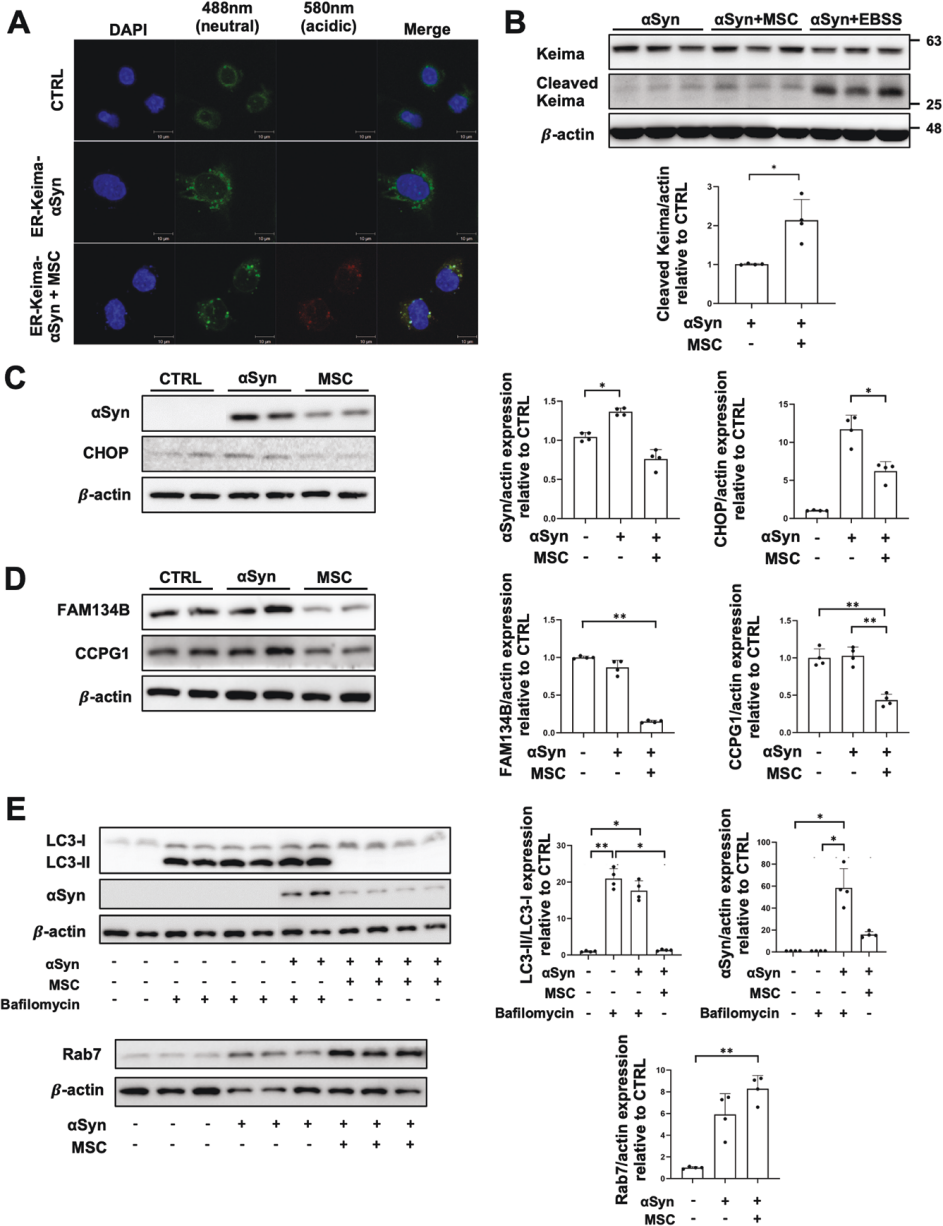
MSCs promote the clearance of αSyn accumulated in the ER and attenuate ER stress through modulating selective ER-phagy. (A) Confocal microscopy monitoring of Keima protein in control, ER-αSyn, and co-culture with MSCs groups in PC12 cells. Scale bar, 10 μm. (B) Western blot analysis for Keima and lysosomal compartments in ER-αSyn, co-culture with MSCs, and EBSS-induced starvation groups (*n* = 4 per group). Quantification of cleaved Keima western blot analysis. (C) Western blot analysis for αSyn and CHOP in control, ER-αSyn, and co-culture with MSCs groups (*n* = 4 per group). Quantification of αSyn and CHOP western blot analysis. (D) Western blot analysis for FAM134B and CCPG1 in control, ER-αSyn, and co-culture with MSCs groups (*n* = 4 per group). Quantification of FAM134B and CCPG1 western blot analysis. (E) Western blot analysis for LC3 II, αSyn, and Rab7 in control, ER-αSyn, co-culture with MSCs, and bafilomycin-treated groups (*n* = 4 per group). Quantification of LC3 II, αSyn, and Rab7 western blot analysis. Differences among conditions were evaluated by Mann-Whitney U test (B) and Kruskal-Wallis with Dunn’s test (C-E). Data are presented as mean ± SE. *P < .05, **P < .01.

Keima, a fluorescent protein whose excitation spectrum changes with pH, allows monitoring the conversion of an autophagosome to an autolysosome through color change.^37^ To determine whether αSyn clearance occurred via the selective ER-phagy pathway, rather than general autophagy, we cloned αSyn into a vector containing the ER retention signal sequence with Keima protein (ER-Keima-αSyn) and transfected PC12 cells with the ER-Keima-αSyn plasmid. Confocal imaging showed that co-culture with MSCs induced ER-phagy (Figure 2A).

Western blot analysis under EBSS-induced starvation conditions, used as a positive control for ER-phagy induction,^38^ showed that co-culture with MSCs for 48 h significantly increased the cleaved form of Keima compared to the αSyn group, indicating that MSCs promoted selective ER-phagy (Figure 2A and B). The level of CHOP, an ER stress marker, was increased in the αSyn group compared to controls. However, co-culture with MSCs for 48 hours successfully attenuated CHOP expression, which was accompanied by decreased αSyn expression (Figure 2C). To identify the ER-phagy receptor involved in αSyn clearance, we analyzed the expression levels of several ER-phagy receptors, including FAM134B, CCPG1, SEC62, and TEX264. No significant difference was observed in the expression levels of SEC62 and TEX264 (data not shown). The expression level of FAM134B was decreased in the αSyn overexpressing group compared to controls. MSC co-culture for 48 h further reduced FAM134B expression. No significant difference was observed in CCPG1 levels between the control and αSyn groups, but the MSC co-culture group showed a significantly decreased CCPG1 level (Figure 2D). We examined autophagy flux by assessing LC3 II and Rab7. The autophagosome marker LC3 II was already decreased in both the αSyn and MSC cocultured groups. Similarly, Rab7, a late endosome marker, was increased in both the αSyn and MSC cocultured groups compared to the control group (Figure 2E). Based on these data, we concluded that a 48-hours MSC co-culture effectively degraded ER-accumulated αSyn via the ER-phagy receptor FAM134B, indicating the endpoint of the autophagy process.

### MSCs promote degradation of αSyn accumulated in the ER through FAM134B-mediated ER-phagy

**Figure 3.**
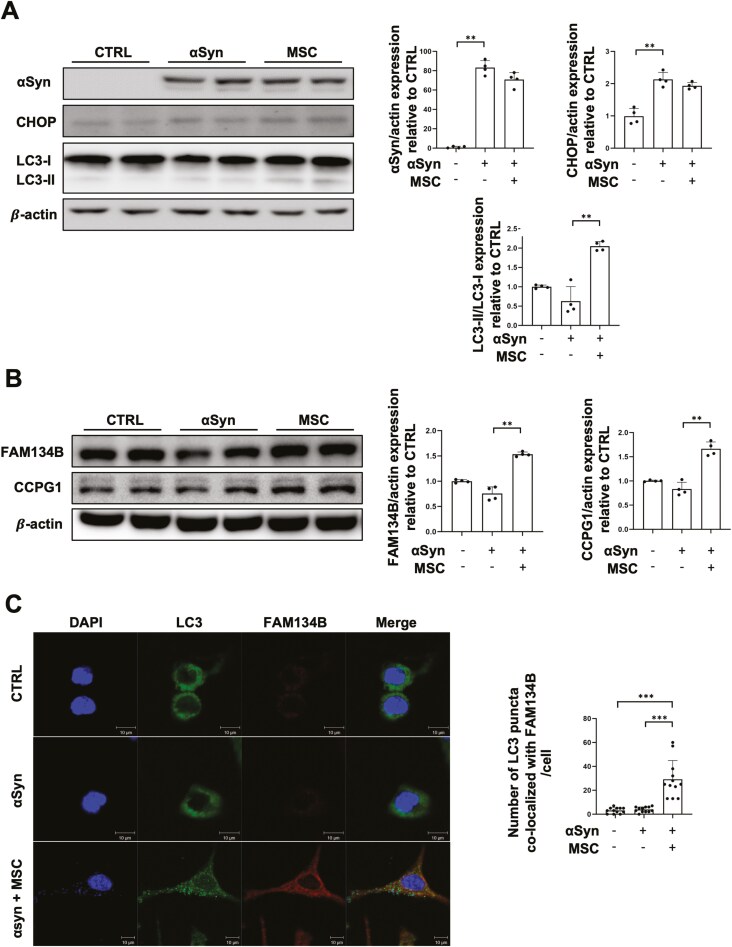
MSCs promote the degradation of αSyn accumulated in the ER through FAM134B-mediated ER-phagy. (A) Western blot analysis for αSyn and LC3 II in control, ER-αSyn, and co-culture with MSCs groups (*n* = 4 per group). Quantification of αSyn and LC3 II western blot analysis. (B) Western blot analysis for FAM134B and CCPG1 in control, ER-αSyn, and co-culture with MSCs groups (*n* = 4 per group). Quantification of FAM134B and CCPG1 western blot analysis. (C) Immunostaining for LC3 puncta and FAM134B co-localization in control, ER-αSyn, and co-culture with MSCs groups. Scale bar, 10 μm. Quantification of LC3 puncta number. Differences among conditions were evaluated by Kruskal-Wallis with Dunn’s test. Data are presented as mean ± SE. **P < .01, ***P < .001.

Since coculture with MSCs for 48 hours completely degraded αSyn accumulated in the ER, we investigated earlier time points to monitor changes in ER-phagy receptors. Coculture with MSCs for 6 hours slightly degraded αSyn in the ER, but there was no significant difference compared to the αSyn group. Additionally, there was no significant difference in CHOP expression between the ER-αSyn group and the MSC coculture group. LC3 II levels were significantly increased in the MSC coculture group compared to the αSyn group (Figure 3A). Under these conditions, there were no significant differences in the FAM134B and CCPG1 expression between the control and αSyn groups, but MSCs elevated FAM134B and CCPG1 levels relative to the αSyn group (Figure 3B). Immunofluorescence staining showed that coculture with MSCs for 6 hours led to increased co-localization of LC3 puncta with FAM134B (Figure 3C). These results suggest that MSCs may regulate FAM134B-mediated ER-phagy early on, with αSyn clearance by ER-phagy occurring later.

### Enhancement of ER-phagy by MSCs has neuroprotective effects and improves motor deficits in the AAV-ER-αSyn model

**Figure 4.**
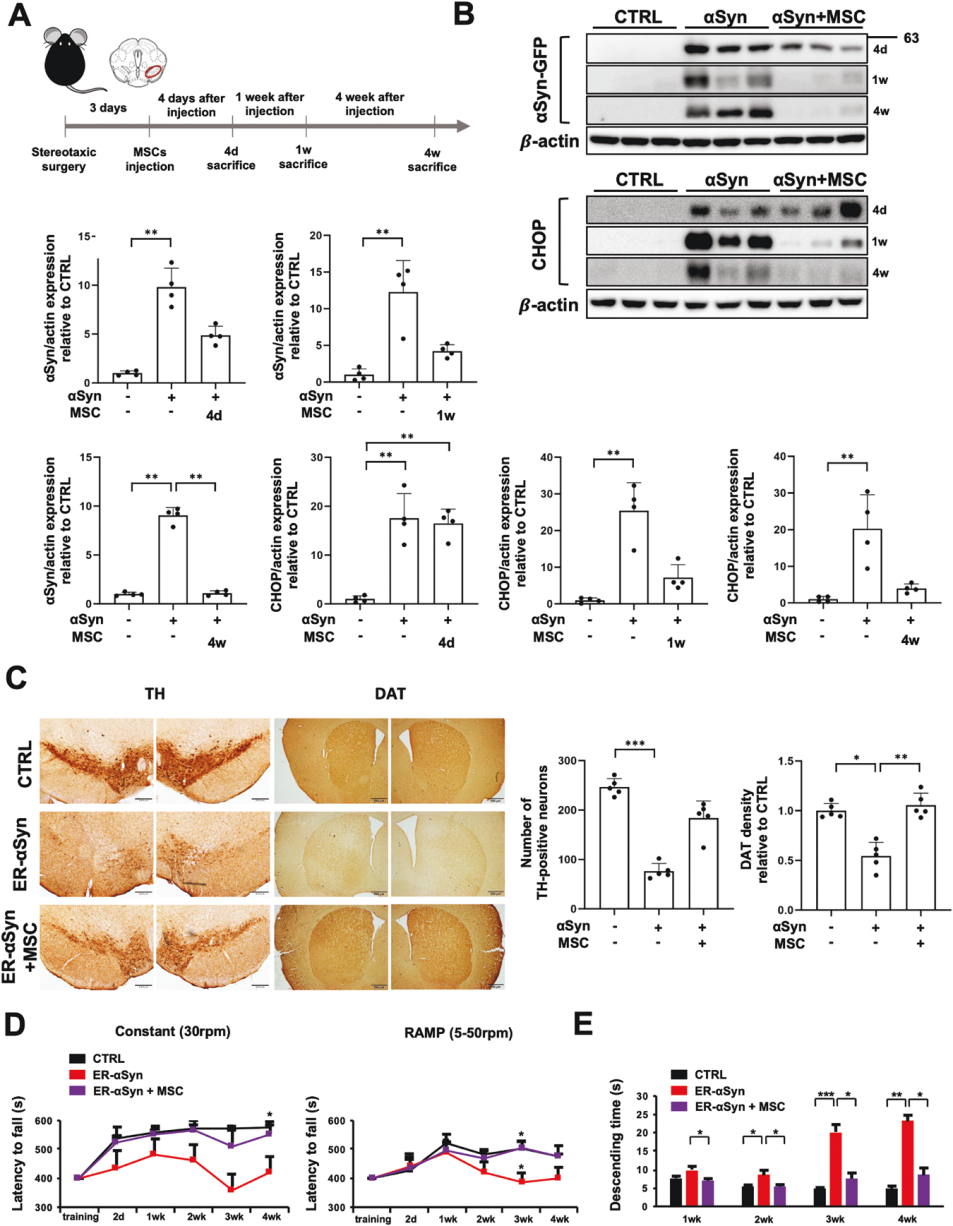
Enhancement of ER-phagy by MSCs has a neuroprotective effect and improves motor deficits in the AAV-ER-αSyn model. (A) Schematic illustrations and experimental design schedule. (B) Western blot analysis for αSyn and CHOP in control, ER-αSyn virus, and MSC-treated groups (*n* = 4 per group). Quantification of αSyn and CHOP western blot analysis. (C) Immunostaining of TH-positive cells in the SN and DAT-positive cells in the ST in control, ER-αSyn virus, and MSC-treated groups. Scale bar, 500 μm. Quantification of TH-positive cell numbers and DAT density (*n* = 5 per group). (D) Behavioral analysis of latency to fall in the rotarod test in control, ER-αSyn virus, and MSC-treated groups (*n* = 9 per group). Significance between ER-αSyn only group and MSC-treated group is presented. (E) Behavioral analysis of the pole test in control, ER-αSyn virus, and MSC-treated groups. Differences among conditions were evaluated by Kruskal-Wallis with Dunn’s test. Data are presented as mean ± SE. *P < .05, **P < .01, ***P < .001.

To examine the effects of MSC-modulated ER-phagy in animal models, we constructed viral vectors overexpressing αSyn in the ER (ER-αSyn virus), confirmed to specifically overexpress αSyn in the ER in our previous study.^23^ The virus was directly injected into the SN region of mice, followed by tail vein injection of MSCs. The in vivo experimental design is illustrated (Figure 4A). We confirmed ER-αSyn expression in the midbrain tissue of mice (Supplementary Figure 1). Western blot analysis 4 d after MSC administration in ER-αSyn mice showed about a 50% reduction in αSyn expression compared to αSyn-only mice. Protein analysis at 4-week post-MSC administration showed a marked decrease in αSyn levels. CHOP expression was significantly increased in ER-αSyn mice compared to control mice, and CHOP expression was decreased 1 and 4 weeks after MSC treatment compared to the ER-αSyn group (Figure 4B).

Further, we examined whether enhanced ER-phagy by MSCs would affect dopaminergic neurons in the SN and presynaptic neurons in the ST. Mice injected with ER-αSyn viruses in the SN showed a significant decrease in TH-positive cells in the SN and presynaptic dopaminergic neurons in the ST compared to control mice. However, mice sacrificed four weeks after MSC administration showed successful recovery of both nigral dopaminergic neurons and striatal presynaptic dopaminergic neurons, with DAT staining density comparable to control mice four weeks after MSC administration (Figure 4C). Behavioral analysis showed that ER-αSyn virus inoculation led to progressively decreased latency to fall times on the Rotarod test compared to control mice. However, MSC treatment in ER-αSyn mice significantly improved performance at 3 weeks and 4 weeks at the ramp and constant testing on the Rotarod, respectively (Figure 4D). Additionally, in the pole test, control mice descended the pole stably using all four paws, while virus-injected mice showed impaired hind limb use, slid, and some even fell from the pole. MSC administration improved the posture and stability of mice while descending from the pole (Figure 4E).

### MSCs promote FAM134B-mediated ER-phagy in AAV-ER-αSyn model

**Figure 5.**
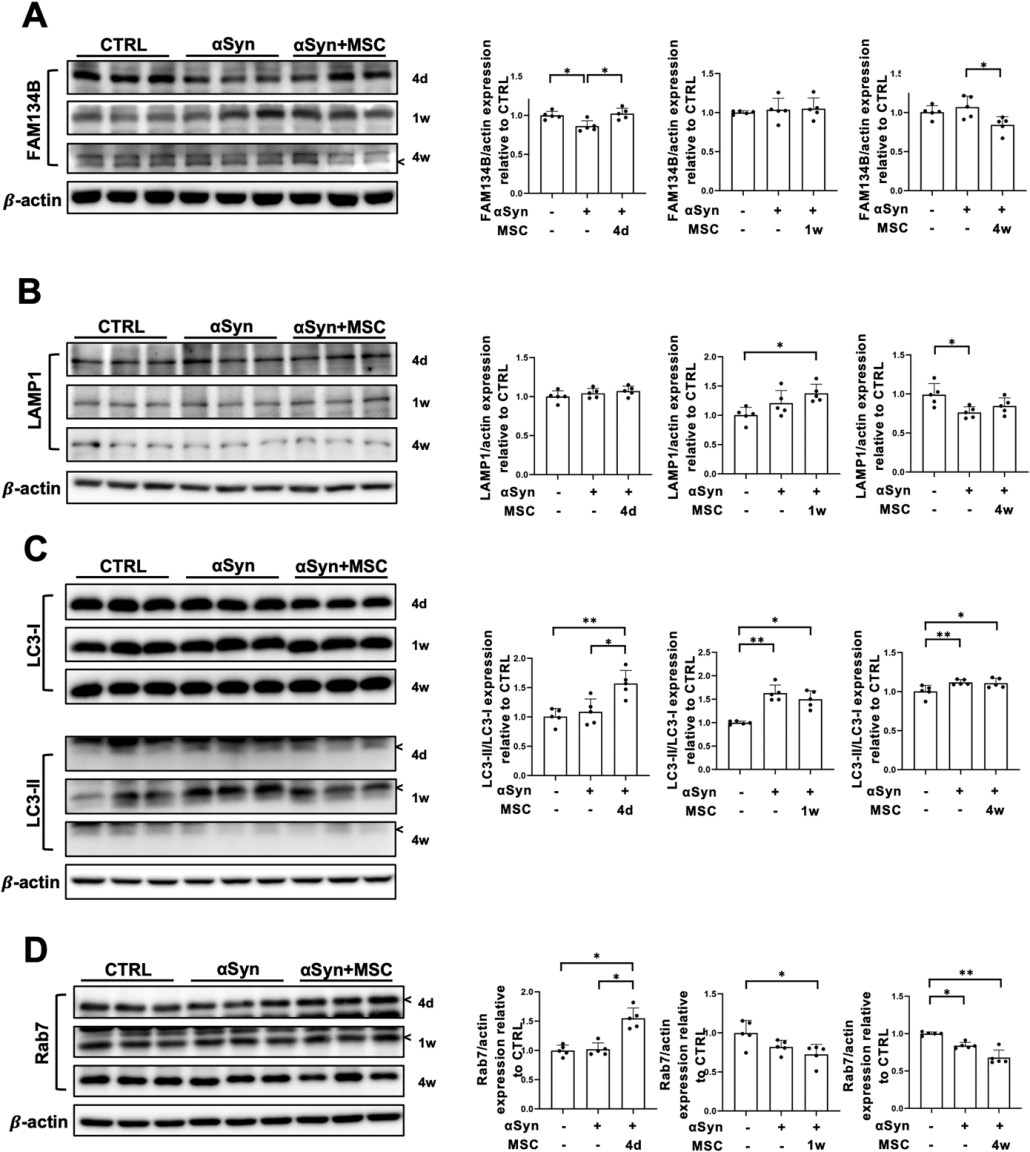
MSCs promote FAM134B-mediated ER-phagy in the AAV-ER-αSyn model. (A) Western blot analysis for FAM134B in control, ER-αSyn virus, and MSC-treated groups (*n* = 5 per group). Quantification of FAM134B western blot analysis. (B) Western blot analysis for LAMP1 in control, ER-αSyn virus, and MSC-treated groups (*n* = 5 per group). Quantification of LAMP1 western blot analysis. (C) Western blot analysis for LC3 in control, ER-αSyn virus, and MSC-treated groups (*n* = 5 per group). Quantification of LC3 western blot analysis. (D) Western blot analysis for Rab7 in control, ER-αSyn virus, and MSC-treated groups (*n* = 5 per group). Quantification of Rab7 western blot analysis. Differences among conditions were evaluated by Kruskal-Wallis with Dunn’s test. Data are presented as mean ± SE. *P < .05, **P < .01.

To determine whether MSCs promote selective ER-phagy, we analyzed ER-phagy-related proteins in the AAV-ER-αSyn animal model. Western blot analysis four days after MSC administration in ER-αSyn mice showed an increased expression of FAM134B compared to αSyn-only mice, reaching levels comparable to the control group. These results are consistent with the in vitro 6-hours coculture data. No significant difference was observed in FAM134B expression in animals sacrificed at one week. However, mice sacrificed 4 weeks after MSC administration unexpectedly showed decreased FAM134B expression compared to the ER-αSyn group (Figure 5A).

To investigate the autophagy flux over time, we analyzed autophagy markers such as LAMP1, LC3, and the late endosome marker Rab7. The expression of LAMP1 showed no change among the groups at 4 days. However, 1 week after MSC administration, ER-αSyn mice showed elevated LAMP1 expression compared to control mice (Figure 5B). These data suggest that FAM134B expression increases at earlier time points, followed by LAMP1 elevation later on.

Further, we analyzed LC3 protein to monitor autophagy flux. Four days after MSC administration, LC3 II expression significantly increased compared to ER-αSyn and control mice, lasting until the one-week post-injection (wpi) time point. However, one week after ER-αSyn injection, mice showed increased LC3 II protein as well, which may represent a cellular defense mechanism against misfolded protein accumulation, and it was continued to the four-week post-injection time point (Figure 5C). The late endosome marker Rab7 was already increased 4 days after MSC administration compared to the ER-αSyn and control mice. Rab7 expression decreased over time, with significant difference at 1-week post-injection. Four weeks after ER-αSyn injection, Rab7 expression decreased compared to control mice, with MSC-treated groups showing even less expression than the ER-αSyn group, correlating with FAM134B expression data (Figure 5D).

### MSCs promote FAM134B-mediated ER-phagy by modulating transcription factor NR4A1

**Figure 6.**
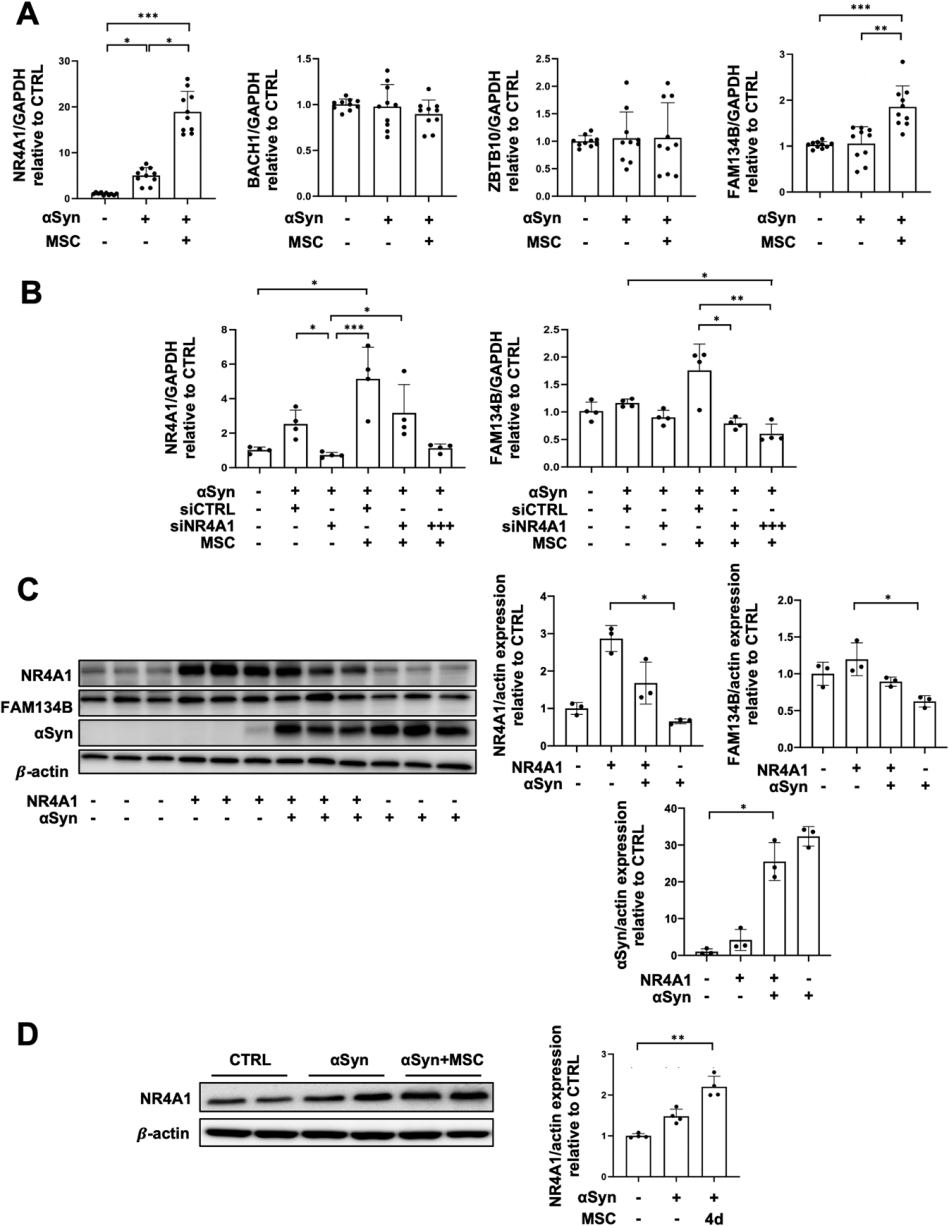
MSCs promote FAM134B-mediated ER-phagy through modulating transcription factor NR4A1. (A) Quantitative real-time PCR of NR4A1 and FAM134B in control, ER-αSyn virus, and MSC-treated groups (*n* = 10 per group). (B) Quantitative real-time PCR of NR4A1 and FAM134B in control/control siRNA, ER-αSyn/NR4A1 siRNA, co-culture with MSCs/control siRNA, co-culture with MSCs/NR4A1 siRNA (single), and co-culture with MSCs/NR4A1 siRNA (triple) groups (*n* = 4 per group). (C) Western blot analysis for NR4A1, FAM134B, and αSyn in control, NR4A1 overexpressed, NR4A1/ αSyn co-overexpressed, and αSyn overexpressed groups (*n* = 3 per group). Quantification of NR4A1, FAM134B and αSyn western blot analysis. (D) Western blot analysis for NR4A1 in control, ER-αSyn virus, and MSC-treated groups (*n* = 4 per group). Quantification of NR4A1 western blot analysis. Differences among conditions were evaluated by Kruskal-Wallis with Dunn’s test. Data are presented as mean ± SE. *P < .05, **P < .01, ***P < 0.001.

To investigate the underlying mechanism of ER-phagy modulation by MSCs, we screened known FAM134B transcription factors, including myocyte enhancer factor 2D (MEF2D), nuclear receptor subfamily 4 group A member 1 (NR4A1), BTB domain, and CNC homolog 1 (BACH1), and zinc finger and BTB domain containing 10 (ZBTB10).^39^ Among them, the mRNA level of NR4A1 in PC12 cells was significantly elevated with MSC coculture compared to ER-αSyn overexpressing cells, followed by an elevation in FAM134B (Figure 6A). Next, using NR4A1 siRNA, we tested whether knockdown of NR4A1 would inhibit the effect of MSCs on ER-phagy enhancement. Control siRNA-treated MSC groups showed increased mRNA levels of FAM134B, whereas NR4A1 siRNA-treated MSC groups showed significantly decreased FAM134B levels. A triple siRNA sequence combination produced a higher knockdown of NR4A1 than single siRNA knockdown, resulting in greater downregulation of FAM134B (Figure 6B). Furthermore, the direct regulatory relationship between NR4A1 and FAM134B, and the subsequent enhancement of αSyn degradation was confirmed (Figure 6C). We also analyzed NR4A1 protein expression in the AAV-ER-αSyn animal model. As expected, four days after MSC administration, there was an elevation in NR4A1 expression compared to ER-αSyn injected mice (Figure 6D). These data suggest that MSCs promote FAM134B-mediated ER-phagy through the regulation of the transcription factor NR4A1 in cellular and murine Parkinsonian models.

## Discussion

This study aimed to elucidate the mechanisms by which MSCs promote selective ER-phagy to clear ER-αSyn, the accumulated αSyn in ER of parkinsonian models. The major findings are: (1) MSCs enhance ER-αSyn clearance and restore ER and cellular function by inducing FAM134B-mediated selective ER-phagy; (2) MSC-enhanced ER-phagy exhibits neuroprotective effects in a PD model with nigral injection of AAV-ER-αSyn; and (3) MSCs regulate ER-phagy through the transcription factor NR4A1. These results suggest that MSCs modulate ER-αSyn via selective ER-phagy, demonstrating neuroprotective properties in PD models.

Several studies have reported that αSyn accumulates in the ER in synucleinopathies,^11^ causing ER stress and dysfunction.^10,11,40-42^ Our previous work also supports the accumulation of αSyn in the ER of patients with PD and αSyn transgenic mice.^23^ Misfolded proteins in the ER are typically processed through either ER-associated degradation (ERAD) or autophagy.^43^ ERAD involves the retranslocation of accumulated proteins into the cytosol for proteasomal degradation, whereas autophagy involves lysosomal degradation.^44,45^ ER stress-induced autophagy can be selective or nonselective. Although many studies have detailed the selective autophagic process and its receptors, specific research on ER-phagy in neurodegenerative diseases such as PD and Alzheimer’s disease (AD) is limited. Our previous study demonstrated that enhancing selective ER-phagy can promote the clearance of ER-αSyn, resulting in neuroprotective effects.^23^

In this study, we evaluated whether MSCs enhance selective ER-phagy in PD models. Ample evidence indicates that MSCs exert neuroprotective effects by secreting neurotropic molecules that modulate the neurodegenerative microenvironment.^46,47^ We previously reported that MSC treatment augments autophagolysosome formation, providing neuroprotective effects in PD^28^ and AD models.^48^ Here, we found that MSCs promote ER-αSyn clearance and reduce ER stress through selective ER-phagy, leading to neuroprotective effects in an AAV-ER-αSyn animal model. When αSyn is overexpressed in the ER, cell viability significantly decreases due to severe ER stress, which is mitigated by MSC co-culture. Using the Keima protein, we confirmed that ER-αSyn was degraded through selective ER-phagy. While MSCs took about 48 h to clear ER-αSyn, the modulation of the ER-phagy receptor FAM134B occurred as early as 6 hours post co-culture. At this early time point, the effects of MSCs on ER-αSyn and CHOP were not evident, implying that receptor modulation is necessary before target protein degradation and subsequent ER stress attenuation. In vivo, AAV-ER-αSyn virus inoculation in the SN caused significant dopaminergic neuron loss and motor deficits. Consistent with in vitro findings, MSC administration increased FAM134B expression 4 d post-injection, cleared ER-αSyn 1-week post-injection, and attenuated CHOP expression 4 weeks post-injection. Dopaminergic neuron loss in the SN and striatal denervation were significantly reduced in MSC-treated animals, resulting in improved performance on the rotarod and pole tests. Both cellular and animal data imply that: (1) misfolded protein accumulation in the ER triggers ER stress; (2) MSC treatment in ER-αSyn conditions elevates ER receptor expression early on; and (3) target protein is subsequently degraded through ER-phagy, alleviating ER stress.

Although recent studies have identified several ER-phagy receptors, their regulatory mechanisms are not well understood. FAM134B isoform, FAM134B-2, is known to be induced under amino acid starvation and regulated by transcription factors such as MEF2D, NR4A1, BACH1, and ZBTB10.^38^ Among these, NR4A1 was upregulated by MSCs in both our cellular and animal PD models, followed by an increase in FAM134B. Using NR4A1 siRNA, we confirmed that NR4A1 suppression specifically counteracted the effect of MSCs on FAM134B induction in ER-αSyn overexpressing PC12 cells. This modulatory effect of NR4A1 was further supported by in vivo samples, showing that MSC-injected mice expressed higher levels of NR4A1 compared to ER-αSyn mice. Thus, this study provides evidence that MSCs promote FAM134B-mediated ER-phagy to clear αSyn via modulation of the transcription factor NR4A1. NR4A1 is an intracellular transcription factor known to play a key role in inflammatory responses and attenuate oxidative stress in PD models.^49^ Additionally, NR4A1 is elevated in a 6-OHDA-induced PD rat model, while genetic disruption of NR4A1 induces dopaminergic cell loss in the SN.^50^ Although several studies have reported associations between NR4A1 and the dopamine system in PD models, our study is the first to investigate the involvement of NR4A1 in selective ER-phagy. The finding that MSCs enhance NR4A1-regulated and FAM134B-mediated ER-phagy presents a promising therapeutic strategy for PD.

## Conclusions

This study demonstrates that MSCs promote FAM134B-mediated ER-phagy, providing a pro-survival effect in a PD cellular model and a neuroprotective effect in a PD animal model. These findings reveal a novel function of MSCs in modulating FAM134B-mediated ER-phagy, suggesting it as a promising therapeutic strategy for PD.

## Supplementary Material

szaf019_suppl_Supplementary_Material

## Data Availability

The data used to support the findings of this study are available from the corresponding authors upon request.
