# Supplementary material for: Mesenchymal stem cells enhance selective ER-phagy to promote α-synuclein clearance in Parkinson’s disease
Source: Stem Cells Transl Med. 2025 Jun 10;14(6):szaf019. doi: 10.1093/stcltm/szaf019 (PMC12150288; doi:10.1093/stcltm/szaf019)
Supplement: szaf019_suppl_Supplementary_Material [file szaf019_suppl_supplementary_material.zip › SCTM_Supplementary Information.docx]

**Supplementary Information**

**Mesenchymal stem cells enhance selective ER-phagy to promote α-synuclein clearance in Parkinson’s disease**

**MSC-enhanced α-Synuclein clearance via ER-phagy**

Ji Eun Lee, PhD^1†^, Kyu Won Oh, MS^1,2†^, Jin Young Shin, PhD^1,5^, Yeon Ju Kim, PhD^1^, Seung-Jae Lee, PhD^2,3^, Phil Hyu Lee, MD, PhD^1,5*^

^1^Department of Neurology, Yonsei University College of Medicine, Seoul 03722, Korea

^2^Department of Biomedical Sciences, Neuroscience Research Institute, Seoul National University College of Medicine, Seoul 03080, Korea

^3^Convergence Research Center for Dementia, Seoul National University College of Medicine, Seoul 03081;

^4^Neuramedy Co. Ltd., Seoul 04796, Korea.

^5^Department of Biomedical Science, Yonsei University College of Medicine, Seoul 03722, Korea

^†^These authors contributed equally to this work.

***Correspondence:**

Phil Hyu Lee (P.H.L.), MD PhD, Department of Neurology, Yonsei University College of Medicine, Seoul 03722, South Korea, Email: [phlee@yuhs.ac](mailto:phlee@yuhs.ac), Tel: +82-2-2228-1608, Fax: +82-2-2228-1608

**Supplementary figure 1.** Expression of ER-aSyn in the midbrain tissue of mice upon substantia nigral injection of AAV8-ER-aSyn. (A) Western blot analysis for αSyn in control and ER-αSyn virus groups (n = 3 per group). (B) Immunofluorescence for αSyn expression in TH-positive neurons in substantia nigra. Scale bar, 50μm. Differences among conditions were evaluated by Mann-Whitney U test. Data are presented as mean ± SE.
