# Supplementary figures and images for: Mesenchymal stem cells enhance selective ER-phagy to promote α-synuclein clearance in Parkinson’s disease
Source: Stem Cells Transl Med. 2025 Jun 10;14(6):szaf019. doi: 10.1093/stcltm/szaf019 (PMC12150288; doi:10.1093/stcltm/szaf019)

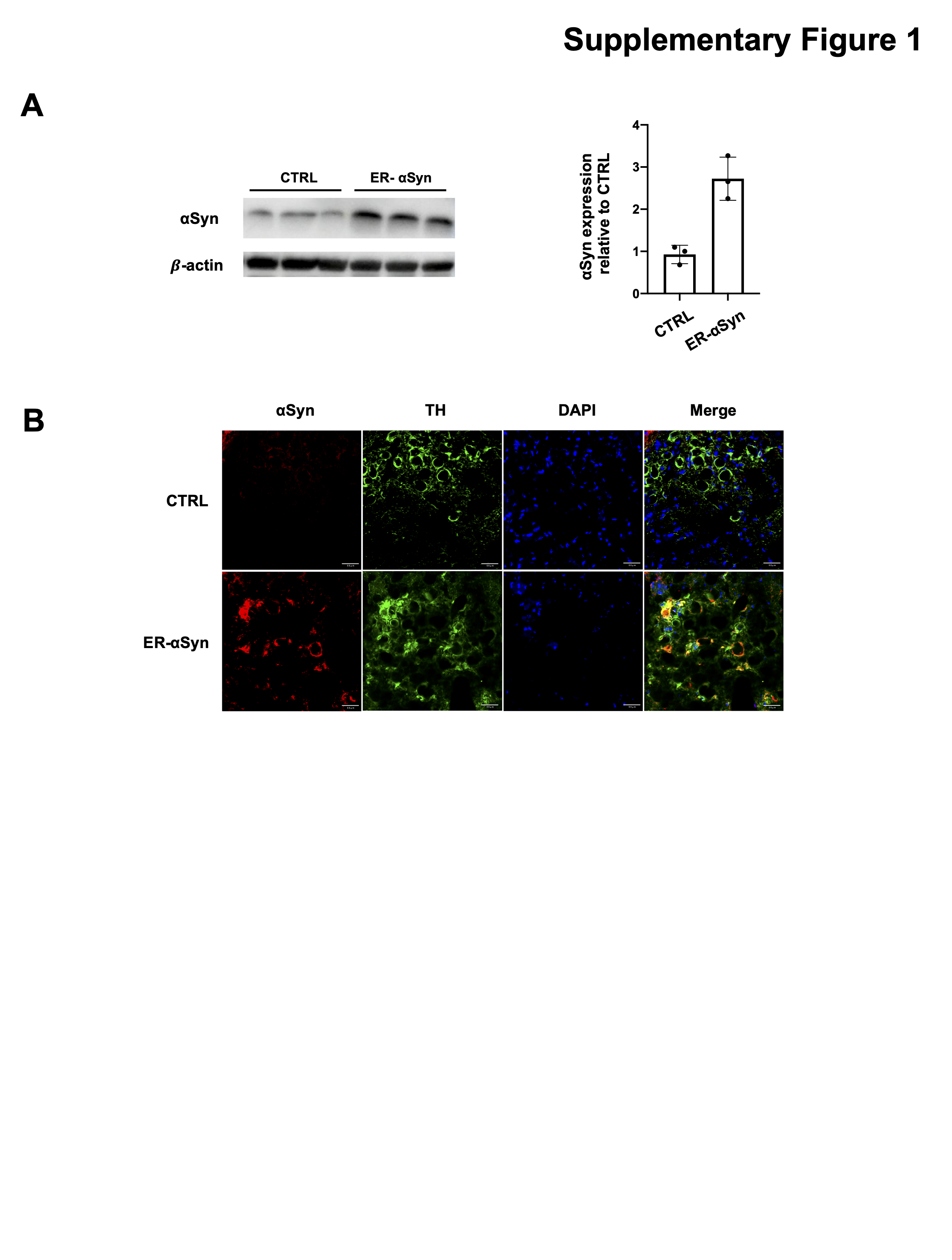

Supplement: szaf019_suppl_Supplementary_Material [file szaf019_suppl_supplementary_material.zip › SCTM_Supplementary figure1.tiff]
